# Supplementary material for: Extended Prescribing Roles for Pharmacists in Poland—A Survey Study
Source: Int J Environ Res Public Health. 2022 Jan 31;19(3):1648. doi: 10.3390/ijerph19031648 (PMC8834828; doi:10.3390/ijerph19031648)
Supplement: Supplementary file 1 [file ijerph-19-01648-s001.zip › ijerph-1533456-supplementary.pdf]

RESEARCH QUESTIONNAIRE

1. After the change in pharmaceutical prescribing rules (01.04.2020), have you issued such a fully-paid prescription to a patient at a pharmacy?
   - Yes
   - No
   - I don't remember

2. Did you issue a *pro auctore* prescription?
   - Yes
   - No
   - I don't remember

If "yes", - please answer the following questions, if "no", "I don't remember" - please go to question 5.

3. If you issued a *pro auctore* prescription, was it a reimbursement prescription?
   - Yes
   - No
   - I don't remember

4. If you issued a *pro auctore* prescription, was it a fully-paid prescription?
   - Yes
   - No
   - I don't remember

5. Did you issue a *pro familiae* prescription?
   - Yes
   - No
   - I don't remember

If "yes" - please answer the following questions, if "no", "I don't remember" - please go to question 8.

6. If you issued a *pro familiae* prescription, was it a reimbursement prescription?
   - Yes
   - No
   - I don't remember

7. If you issued a *pro familiae* prescription, was it a fully-paid prescription?
   - Yes
   - No
   - I don't remember

8. Can you give an estimate of the average number of prescriptions you issued in the last month (including *pro auctore* and *pro familiae* prescriptions)?
   - 0
   - 1 - 5
   - 5 - 10
   - 10 - 15
   - 15 - 20
   - More than 20

9. Considering the prescriptions issued - both for patients and those for yourself or your family members - what was the most common reason for it?
   - repeat prescriptions (renewal of a doctor's order, treatment for chronic diseases)
   - prescriptions for life-saving drugs (e.g. epinephrine, insulin)
   - based on own diagnosis - in the case of a minor illness
   - at the request of patient/family member
   - other reason

10. In your opinion, should a pharmacist be able to issue reimbursement pharmaceutical prescriptions for patients?
    - Yes
    - No

- I have no opinion

11. In your opinion, should a pharmacist be able to issue pharmaceutical prescriptions as well as *pro auctore* and *pro familiae* prescriptions - for magistral drugs?
- Yes
- No
- I have no opinion

12. Do you think the current rules governing pharmaceutical prescriptions are clear and transparent?
- Definitely not
- Rather not
- Difficult to say
- Rather yes
- Definitely yes

13. Do you feel that there is abuse of prescriptions by pharmacists?
- Yes
- No
- I don’t know

14. Do you feel anxious about your ability to write prescriptions?
- Yes
- No
- I don’t know

15. If "yes", what does your anxiety concern:
- The possibility of harm to the patient as a result of a misjudgment
- Problems with reimbursement (NHF)
- Uncertainties in the current legislation
- Other reason: ...

Metrics

16. Professional status:
- Master of pharmacy in a community pharmacy
- Manager in a community pharmacy
- Master of pharmacy in a hospital pharmacy
- Manager of a hospital pharmacy
- Job other than in a pharmacy, but related to the profession of pharmacist

17. Professional experience (years)
- Less than 5
- 5 - 10
- 10 - 20
- More than 20

18. Place of pharmacist's professional activity
- Individual community pharmacy (not part of a pharmacy chain)
- Network community pharmacy (small network: several pharmacies)
- Network community pharmacy (large network: dozens/over 100 pharmacies)
- Hospital pharmacy
- Other: ....

19. Pharmacy location/workplace:
- City of up to 10,000 inhabitants
- Locality with 11 - 50 thousand inhabitants
- City with 51 - 200 thousand inhabitants
- City with more than 200 thousand inhabitants
- City with more than 200 thousand inhabitants
